# Can NF-κB Be Considered a Valid Drug Target in Neoplastic Diseases? Our Point of View

**DOI:** 10.3390/ijms21093070

**Published:** 2020-04-27

**Authors:** Manuela Labbozzetta, Monica Notarbartolo, Paola Poma

**Affiliations:** Department of Biological, Chemical and Pharmaceutical Science and Technology (STEBICEF), University of Palermo, 90128 Palermo, Italy; manuela.labbozzetta@unipa.it (M.L.); monica.notarbartolo@unipa.it (M.N.)

**Keywords:** MDR, cancer, NF-κB, drug target

## Abstract

Multidrug resistance (MDR), of the innate and acquired types, is one of major problems in treating tumor diseases with a good chance of success. In this review, we examine the key role of nuclear factor-kappa B (NF-κB) to induce MDR in three tumor models characterized precisely by innate or acquired MDR, in particular triple negative breast cancer (TNBC), hepatocellular carcinoma (HCC), and acute myeloid leukemia (AML). We also present different pharmacological approaches that our group have employed to reduce the expression/activation of this transcriptional factor and thus to restore chemo-sensitivity. Finally, we examine the latest scientific evidence found by other groups, the most significant clinical trials regarding NF-κB, and new perspectives on the possibility to consider this transcriptional factor a valid drug target in neoplastic diseases.

## 1. Introduction

Inducible transcription factors are responsible for regulating immediate and long-lasting cellular responses that are indispensable for the adaptation of organisms to environmental variability. A transcription factor that serves as a key to the response to changes in the environment is nuclear factor-kappa B (NF-κB), which plays a crucial role in many biological processes. In mammalian cells, NF-κB is a dimeric transcription factor formed by five members of a family of proteins: RelA (p65), RelB, c-Rel, p50/p105 (NF-κB1), and p52/p100 (NF-κB2) [1]. The homo and heterodimer induction of these five members can be regulated by various signals such as those of bacteria, cytokines, viral expression, growth factors, and stress stimuli [2]. In the non-stimulated cells, these members exist and are linked to the IκB kinase (IKK) family which includes three subunits: IKKα, IKKβ, and the NF-κB essential modulator (NEMO). IKKα and IKKβ are catalytic kinases, while NEMO serves as a scaffold component for the IKK complex. Generally, the IκB proteins sequester the interacting NF-κB dimeric complexes in the cytoplasm, thereby maintaining NF-κB in an inactive state; therefore, the ability of NF-κB to translocate to the nucleus, bind DNA, and regulate gene expression is blocked. There are two distinct pathways that lead to the activation of NF-κB—the canonical and non-canonical pathways. In the canonical pathway, the IKK complex requires IKKα, IKKβ, and NEMO to activate the induction of complexes such as p50/RelA-IκBα, while the non-canonical pathway only requires IKKα and induces the partial degradation of p100/RelB to p52/RelB. After stimulation, the IKK complex phosphorylates the IκB proteins, which undergoes proteasome-mediated degradation. This determines the release of NF-κB complexes and allows for their interaction in the nucleus with target DNA sequences, regulating the expression of more than 200 genes implicated in cell survival/apoptosis, cell growth, immune response, and inflammation [3]. The dysregulation of this pathway has been observed in many diseases, including cancer. The study of NF-κB in cancer development started when several members of the NF-κB protein family were found to be mutated in certain types of neoplastic diseases, especially those with hematopoietic origins, thus suggesting a link between NF-κB and oncogenesis. Initially identified for its role in the immune system and hematopoietic cancers, c-Rel has increasingly been associated with solid tumors and other pathologies. c-Rel is frequently amplified in Hodgkin’s lymphomas, in large B-cell lymphomas, and in some follicular and mediastinal B cell lymphomas. Constitutively active NF-κB has been detected in malignant cells derived from patients with multiple myeloma, acute myeloid leukemia, acute lymphocytic leukemia, and chronic myeloid leukemia [4,5]. The constitutive or aberrant activation of NF-κB is also often found in a variety of solid tumors including ovarian cancer [6], prostate cancer [7], gastric carcinoma [8], colorectal cancer [9], hepatocellular carcinoma, [10] breast cancer [11], and triple negative breast cancer [12]. The mechanism of such persistent NF-κB activation is not clear but may involve defects in signaling pathways, mutations, or chromosomal rearrangements, and it is almost always associated with a resistant phenotype and an unfavorable prognosis [13,14,15]. The development of resistance to chemotherapy, especially in its multiple form (multidrug resistance, MDR), is the main limitation of successful treatment in tumors. The transactivating forms of NF-κB (e.g., dimers containing the p65/RelA subunit) may up-regulate the expression of several genes involved in anti-apoptosis, cell proliferation and invasion, and drug resistance (e.g., IAPs, Bcl-2, Bcl-X_L_, cyclin D1, c-myc, interleukin-6 (IL-6), cyclooxygenase-2 (COX-2), inducible NO-synthase (iNOS), matrix metalloproteinases (MMPs), and MDR1/P-gp) [16]. This review highlights our observations on the role of NF-κB in neoplastic disease, especially in multi-drug resistant models, and shows the different approaches considered to modulate its expression and its activation. The suppression of constitutive NF-κB activation inhibits the oncogenic potential of transformed cells and thus makes NF-κB an interesting new therapeutic target in cancer.

## 2. NF-κB and Oncogenesis

The role of NF-κB in human cancer has attracted attention. NF-κB regulates many genes involved in the promotion of cancer (e.g., those of clonal expansion, cell proliferation, diversification, angiogenesis, adhesion, extravasation, and the degradation of extracellular matrix). Several studies have shown how NF-κB is capable of modulating the expression of pro-inflammatory genes like tumor necrosis factor (TNF), IL-1, iNOS, MMP-9, and many other chemokines. Furthermore, the activation of NF-κB may regulate the production of prostaglandins via the gene COX2, which has been shown to be overexpressed in a variety of cancers [17]. In tumor tissues with elevated NF-κB activity, the accumulation of pro-inflammatory cytokines at the tumor site directly contributes to the pro-tumorigenic microenvironment. Chronic inflammation can lead to genomic instability and genetic mutations that favor tumor initiation and development [18]. Additionally, cancer cell growth can be promoted by activating mutations in transcription factors, such as β-catenin, which controls expression of genes that stimulate proliferation [19]. Many studies have suggested that NF-κB controls the anti-apoptotic mechanisms associated with oncogenesis. NF-κB induces the expression of anti-apoptotic genes such the FLICE inhibitory protein (FLIP), the inhibitor of apoptosis proteins c-IAP1/2, Survivin, and XIAP (IAPs), and the members of the family of Bcl-2 apoptosis regulators that are often overexpressed in human tumors. NF-κB activation variably controls the regulation of cell cycle proteins (e.g., cyclin D1 and c-myc) and the interaction with various cellular components (e.g., p300 and p53) that promote or induce apoptosis [20,21]. NF-κB has been reported to promote both angiogenesis and metastasis in certain tumor models, potentially through the regulation of vascular endothelial growth factor (VEGF) and MMPs [22,23]. Its activation not only promotes the proliferation of cancer cells, suppresses apoptosis, and attracts angiogenesis, it also induces epithelial mesenchymal transition [24,25], which facilitates distant metastasis. In fact, the activation of NF-κB also seems to be part of a regulatory loop involving MDA-9/Syntenin, a scaffolding PDZ domain-containing protein overexpressed in multiple human cancers cell lines, that functions as a positive regulator of tumor cell invasion and metastatic spread [26,27,28,29,30]. Finally, NF-κB activation has been shown to induce resistance to various chemotherapeutic agents. NF-κB participates in several phases involved in drug resistance processes in particular, it modifies the expression of the *mdr*1 gene whose product, P-glycoprotein (P-gp), is a transporter of plasma membranes that induces the outflow of chemotherapy, not only in tumor induction and progression but also in resistance to therapy [16,31,32]. Since expression of the multidrug transporter P-gp was found to be NF-κB-dependent, it is believed that NF-κB inhibitors can decrease P-gp expression and restore chemo-sensitivity [33,34].

## 3. NF-κB in Multi-Drug Resistant Human Tumor

The development of MDR to chemotherapy remains a major difficulty in the treatment of cancer. MDR is multifactorial, and pleiotropic cellular signals are simultaneously involved in this process. MDR can be intrinsic or acquired. In intrinsic resistance, the cancer cells are inherently resistant or unresponsive to therapeutics. In acquired resistance, cancer cells that were initially responsive become unresponsive during the course of treatment. The mechanisms responsible of MDR are multiple and the most studied are related with the overexpression of several efflux membrane proteins. Among them, the ATP-binding-cassette (ABC) superfamily largely contributes to MDR, resulting in the increased translocation of the cytotoxic drugs out of the cell, consequently reducing their intracellular concentrations and their biological effect [35,36].

In this review we will examine in particular the role of NF-κB in some human tumor models such as triple negative breast cancer (TNBC), hepatocellular carcinoma (HCC) and acute myeloid leukemia (AML) characterized by high aggressiveness and little responsiveness to drug treatments.

Breast cancer (BC) is a complex disease entity with different biological characteristics and clinical behavior. Many features like age, tumor size, axillary node involvement, angio-lymphatic invasion, histological grade, hormonal receptor status (estrogen and progesterone) and HER-2/neu expression, have been defined to predict treatment response and outcome in breast cancer [37]. In particular, breast cancer cells called TNBC are characterized by lack of estrogen, progesterone and HER-2/neu receptors [38]. TNBCs account for ∼20% of all breast cancers and are associated with epithelial-to-mesenchymal transition (EMT) and a high propensity for early metastasis and risk of mortality [39]. TNBC is often, but not always, a basal-like breast cancer and the presence of stem cells from breast cancer contributes to the onset of cancer and resistance to chemotherapy [40,41]. There is increasing evidence that aberrant activation of NF-κB signaling is a frequent characteristic of TNBC cells, although the underlying causes of this activation have remained largely elusive [42,43]. Studies from gene expression profiling analysis revealed that the nuclear factor NF-κB pathway has been strongly implicated in the pathogenesis of certain TNBCs [44,45], where it affects cell proliferation, suppresses apoptosis as well as promotes anchorage-independent growth [46]. Furthermore, the breast tumor subtypes exhibit different forms of NF-κB activation, some associated with enhanced p50/RelA DNA binding, some with enhanced p50 or p52 DNA binding activity, some with enhanced c-Rel expression. Activated NF-κB (p50/RelA) was detected predominantly in ER-negative vs. ER-positive breast tumors and mostly in ER-negative and ErbB2-positive tumors (86%) [47]. Lee et al. [48] posited a role of enhancer of zeste homolog 2 (EZH2) in the constitutive activation of the NF-κB target gene expression in ER-negative basal-like breast cancer cells. This proposed function of EZH2 requires the physical interaction with RelA/RelB to promote the expression of NF-κB targets [48,49]. In the past, our group had obtained tumor cell lines with characteristic of multi-drug resistance acquired, treating the wild-type cells with gradually increasing concentrations of doxorubicin, chemotherapeutic drug belonging of DNA intercalators and topoisomerase II inhibitors. In particular, MCF-7R is the variant of a breast cancer cell line MCF-7. Our studies have shown that the MCF-7R cell line is characterized by different mechanisms of drug resistance as the lack of estrogen receptor α (ER α) and the overexpression of NF-κB, P-gp, different IAPs and COX-2 [50].

Furthermore, we have also highlighted how the NF-κB transcription factor (p50/p65) is constitutively active in three TNBC cell lines with different characteristics, the SUM 149, the SUM 159 and the MDA-MB-231 [46,51]. The ability of NF-κB to react to different stimuli and the capacity to form different homo- and hetero-dimers can explain its involvement in biological systems with different phenotypic traits. In fact, SUM 149 derived from inflammatory breast cancer (IBC), a basal-like subtype, SUM 159, a stem-like subtype while MDA-MB-231 is claudin-low subtype [52]. Today again there are no treatment guidelines for TNBC because it appears highly aggressive and not responsive to antineoplastic drugs [53,54], so challenges remain to identify potential targets in the field of TNBC research for treating this disease. The transcription factor NF-κB could be a useful target for the treatment of these tumors [55].

The primary liver cancer, HCC, is a frequent tumor, which results in high mortality and most often exhibits a poor response to current drug therapies [56]. In hepatocarcinogenesis, the inception of chronic inflammation, characterized by cell death and subsequent compensatory proliferation that can develop in liver cirrhosis and the presence of altered signal pathways, including the signaling of the nuclear factor κB, represent well known oncogenic factors [57]. The NF-κB pathway constitutes an important link between inflammation and tumorigenesis and has been found to be constitutively active in hepatocellular carcinoma with consequent increase in cell proliferation, drug resistance and invasiveness [58,59]. However, the role of NF-κB in liver cancer is conflicting [60]. Some studies have shown NF-κB’s ability to promote inflammation-associated cancer and inhibition of its signaling pathway in hepatocytes attenuates the onset of HCC associated with inflammation [61,62]. Other authors suggest instead a role as tumor suppressor in hepatocytes since NF-κB inactivation can promote hepatocarcinogenesis and induce spontaneous development of HCC [63]. NF-κB appears to play a role in regulating apoptosis in HCC through the activation of its downstream target genes. These results suggest that pro-apoptotic signals can induce NF-κB, which may in turn induce expression of genes involved in the suppression of apoptotic signals including the IAPs. Furthermore, as it is responsible for the overexpression of P-glycoprotein, it contributes to the creation of drug resistance that characterizes all neoplastic diseases with an unfavorable prognosis such as HCC [64]. Today, in fact, the main strategy to prolong the survival of patients with hepatocellular carcinoma who present advanced disease is chemotherapy, however, the onset of MDR in cancer patients often determines the failure of drug treatment [65]. Our previous study [66] has shown how NF-κB is constitutively activated in an HCC cell line, HA22T/VGH, and its inhibition is helpful in antagonizing IAPs and other target genes implicated in the biology of this tumor. These results suggesting that the NF-κB activities may be important targets for novel therapeutic approaches in the treatment of human HCC.

In addition to solid tumors, NF-κB activation in hematological malignancies has been widely observed [4,5]. AML is a very heterogeneous neoplasm of the hematopoietic stem cell, a malignant clonal disorder characterized by many alterations that inhibit differentiation of cells and induce proliferation or accumulation of blasts, and by low production of healthy hematopoietic cells [67]. Different constitutively activated signaling pathways play critical roles in the survival and growth of acute myeloid leukemia cells. These include NF-κB pathways [68]. Constitutive NF-κB has been detected in more than 50% of cases, enabling leukemic cells to resist apoptosis and stimulate uncontrolled proliferation [69,70,71]. NF-κB activation in myeloid cells typically enhances inflammation in the tumor microenvironment by increasing the secretion of pro-inflammatory cytokines such as TNF-α and IL-6, which eventually leads to rapid proliferation of tumor cells [72] and is responsible for inducible nitric oxide synthase (iNOS) activation to increase nitric oxide (NO). NO production can control cell survival, as acute NO production triggers apoptosis, but conversely, chronic NO production by constitutively active signaling of NF-κB leads to inhibition of the apoptosis mechanism [73]. Moreover, NF-κB is responsible for regulating the expression of heme oxygenase-1 (HO-1), which is a well-known catabolic enzyme for free heme [74]. The upregulation of HO-1 has been reported in AML and contributes to evading TNF-induced apoptosis [75]. As mentioned above, the phosphorylation of NEMO is an essential step in canonical NF-κB pathway activation. In fact, the activation of NF-κB is possible only when NEMO binds to the IKK complex. Since NF-κB has been found constitutively active, both in the blasts and stem cells of acute myeloid leukemia [76], and its activation is detected in the bone marrow of patients with myelodysplastic syndrome (considered a precursor disease of AML [5]), the lack of formation of the NEMO/IKK complex due to the use of NEMO-binding domain peptides could represent a therapeutic strategy to indirectly inhibit NF-κB activation in AML. Additionally, in leukemic cells NF-κB is responsible for the establishment of drug resistance through the control of the expression of the *mdr1* gene [77]. Several studies have shown how among the different cellular mechanisms responsible for drug resistance in patients with acute leukemia and other hematological malignancies, the best characterized is the phenotype of multi-drug resistance mediated by P-gp. The characterization of the HL-60 cell line and its MDR variant HL-60R, obtained in our laboratory following increasing exposures of doxorubicin, showed how the NF-κB factor (p50/p65) is overexpressed in the resistant variant, and it is responsible for up-regulating the expression of the P-gp gene and several members of the IAP family [78,79]. Alterations in IAPs proteins are prevalent in many types of human cancer and are associated with chemoresistance, disease progression, and poor prognosis [80]. HL-60R, in contrast to its parental cells, in fact, lacked sensitivity to cell death induction from diverse stimuli, including doxorubicin and cisplatin administration [77]. By inhibiting NF-κB, it is possible to reduce the expression of these targets so that the sensitivity of therapy can be improved on malignant hematological cells [81,82].

## 4. NF-κB as Molecular Drug Target

For all the reasons listed above, NF-κB can be considered a valid molecular therapeutic target in tumor diseases. NF-κB inhibition, through different approaches from natural and multi-target compounds to synthetic drugs and target therapies, produces the arrest of cell growth and invasive capacity, as well as an increase in the response to anticancer treatments. 

Today, the targeted therapy is certainly the option of therapeutic choice most valued by oncologists, and, with the innumerable products as monoclonal antibodies, tyrosine kinase inhibitors, immunotherapy, it has been imposed on classical antitumor drugs. Unfortunately, there are many aspects of targeted agents to consider including high costs and a relative efficacy in the case of highly unstable tumors, which, from the genetic point of view, strongly mutate in the expression of different proteins. The drug resistance, innate or acquired, poses considerable limits on the effectiveness of single-targeted agents, and, in the last few years, there has been an increase of multi-drug resistance mechanisms in regard to these drugs. Moreover, single-targeted agents are not without important side effects, so in this context, multi-targeted agents are proposed as valid alternatives to molecular therapy because they are able to interfere with different pathways that are simultaneously altered in the cancer cell and because of their limited toxicity.

**Figure 1:**
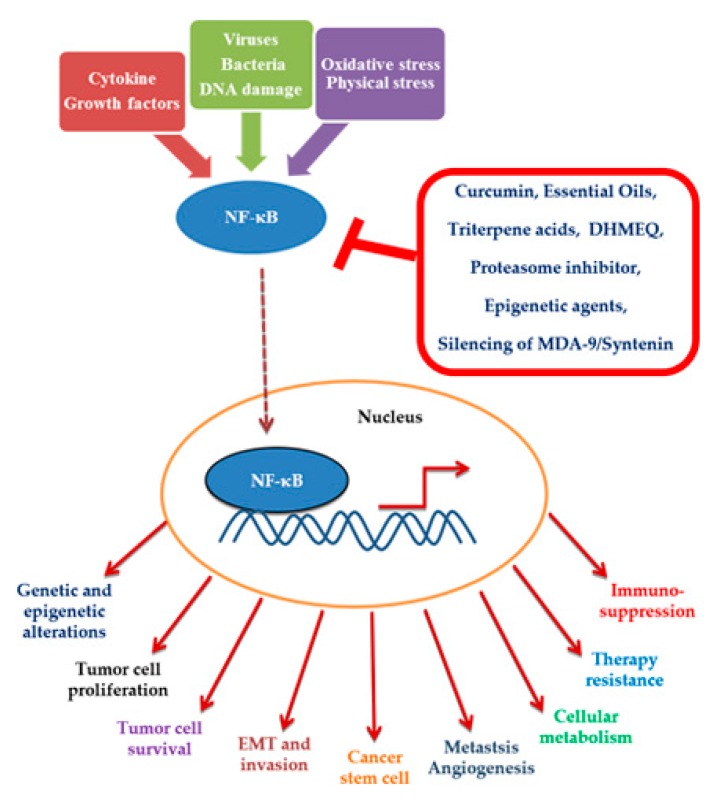
Events following the activation of nuclear factor kappa B (NF-κB) in the neoplastic cell and molecules capable of hindering its activation or translocation to the nucleus.

In this review, we examine all our observations concerning the use of compounds, natural, synthetic, multi-targeted, and single-targeted, that act as pharmacological targets of NF-κB and that were conducted on MDR cancer models (Figure 1).

### 4.1. Natural Compounds

#### 4.1.1. Curcumin

For several years, our group has been analyzing the antitumor properties of curcumin. Curcumin (diferuloylmethane) is a dietary polyphenolic compound extracted from *Curcuma longa L*. and present in curry spice; traditionally, it is used in Indian medicine. Curcumin has many properties like those of anti-inflammatory, anti-oxidant, antimicrobial, and antitumor kinds; in particular, it has exhibited tumor suppressive and preventive activities in many in vitro and in vivo models [83,84]. Curcumin is a “privileged structure” that is able to affect different pathways deregulated in cancers; it suppress the activation and expression of numerous factors responsible for proliferation, drug resistance, and apoptosis inhibition—such as AP-1, MAPK, Akt, COX-2, P-gp and, in particular, NF-κB—through IκB phosphorylation inhibition [85,86].

HCC, as mentioned above, is characterized by constitutive activation of NF-κB and also by an innate MDR. In human HCC cell lines, we observed significant growth inhibitory and apoptotic effects following treatment with curcumin.

In addition, on HA22T/VGH cells, curcumin has been shown to potentiate the antitumor and apoptotic effects of cisplatin, a chemotherapeutic drug belonging to a group of DNA alkylating agents, and the inhibitory effects on NF-κB activation carried were found to lead to a decrease of the expression of some its targets, as IAPs and COX-2 [63].

We observed the same antitumor effects of curcumin in other MDR cancer models.

On MCF-7 and its variant MCF-7R, we analyzed the effects of curcumin and its isoxazole analog, observing that antitumor property of the two compounds, in term of cell proliferation inhibition and death induction, were equal in the MDR model and its parental cell line [50,87]. This result highlighted how curcumin is capable to bypass the molecular mechanisms that make cancer cells resistant to treatment with many anticancer drugs. On the other hand, the same natural compound resulted in a formidable lead compound to synthesis new derivatives with higher antitumor capacities. Curcumin, in fact, is endowed with a diketone function, fundamental to its antitumor activity, to which the ability to form adducts with the –SH groups and generate reactive oxygen species—determining consequently cell death—is attributed [88,89,90]. In the past, we tested the antitumor effects of numerous derivatives obtained by bioisosteric substitutions at the 1,3 dicarbonyl fragment of curcumin with enamino and oxime functions. The isoxazole analog is one of many derivatives that our group have analyzed; it is a more potent form of curcumin (by approximately two-fold) on cell growth inhibition in HA22T/VGH cells, MCF-7, and HL-60, and in the corresponding MDR variant, a dioxime benzyl analog has been revealed as the most active of all in cellular models of HCC and breast cancer. In particular, the MDR variant was able to inhibit cell proliferation and to induce cell death much more than curcumin, and it was able to counteract NF-κB activation [91].

More recently, we analyzed the antitumor effects of curcumin and its new derivatives on two TNBC cell lines, SUM 149 and MDA-MB-231 [51].

Two different approaches have been used in the synthesis of these compounds; as mentioned above in fact, it is well known that the α,β-unsaturated di-keto portion is fundamental for anticancer activity of curcumin, as well as phenolic groups for anti-oxidant capacities, but it is also known that the poor bioavailability and stability of curcumin in physiological media depend on these groups [92,93]. For this motif, we have used analogs in which the phenolic OH groups are completely or partially substituted and an analog with a peculiar structure, obtained by reaction between an electrophilic sulfenic group and the nucleophilic central carbon 1,3-diketone, producing a thioether. In both cases we have observed good anti-proliferative and pro-apoptotic effects on two cellular models examined. In regard to NF-κB, our data once again confirmed that curcumin is a strong inhibitor of NF-κB activation in TNBC, and two of the new analogs keep this property [94].

The antitumor effects of curcumin on acquired multidrug resistance models like MCF-7R cells and HL-60R cells, comparable or stronger respect to parental cell lines, have proven that the compound exerts inhibitory effects on the function of P-gp, as has been reported by numerous authors as well, including us [50,87,95]. Indeed, curcumin is not a substrate of P-gp, but its MDR reversal effects could depend on inhibition of substrate binding or of ABCB1 ATPase activity. We had also speculated an indirect inhibitor mechanism by downregulation of the P-gp gene expression depending by NF-κB inhibition. Unfortunately, on the contrary to what observed by the other authors about the sensitization of MDR tumor cells to established substrates of these efflux pumps, like vinblastine, etoposide, and mitoxantrone [95], we never have observed any reversal activity of P-gp-mediated resistance to doxorubicin by curcumin or its analogs in the MDR models described above [50].

Another important aspect to consider is the limited pharmacological use of curcumin in vivo because of its low solubility in aqueous media and short half-life; consequently, curcumin bioavailability is poor, particularly after oral or topical administration [96,97,98]. In the last few years, we have examined the possible convenience to use designed carrier that could increase the drug delivery and allow for a wider applicability of curcumin in vivo. In this regard, several drug delivery systems (DDS), such as the curcumin loading into liposomes or nanoparticles, have been developed by other groups, though these are not always exploitable for clinic use due to high side effects and low efficacy [99,100,101].

We instead evaluated the anti-proliferative properties of a functionalized halloysite-curcumin system (HTN-curcumin), in which curcumin was covalently linked to halloysite precursor through GSH- or pH-responsive bonds in HCC (HA22T/VGH and Hep3B), TNBC (SUM 149 and MDA-MB-231), and AML cell lines (HL-60 and its variant HL-60R).

The halloysite nanotubes were not toxic towards the cells and enhanced the aqueous solubility of curcumin; moreover, the HTN-curcumin prodrug induced curcumin release from nanoparticles depending by intracellular GSH, and all these characteristics are responsible for the increased cytotoxicity of the HNT-curcumin compared to free curcumin observed in all analyzed cellular models [102,103]. Further investigations are underway to assess whether the advantage of these systems is also true in NF-κB regulation.

#### 4.1.2. Essential Oils

Essential oils (EOs) are mixtures of chemical compounds that constitute the natural endogenous protection of plant species against parasites, bacteria, and fungi; these oils can have important implications in clinics for humans, taking advantage of their anti-inflammatory, antibacterial, anti-mitotic, and anti-proliferative activities [104,105,106,107,108].

It is noteworthy that the oils are highly fat soluble and are characterized by a low toxicity and often by a multi-target activity, precisely because they are composed of several molecules with different pharmacological actions.

Our group assessed the antitumor and antibacterial properties of the essential oils extracted from different plant species, with special focus on their ability to act as inhibitors of NF-κB, a function likely attributable to terpenic compounds of the oils.

A study on the essential oil of *Cyphostemma juttae* [109] showed the cytotoxic effects and pro-oxidant activity of this oil in two TNBC cell lines (MDA-MB-231 and SUM 149). Our results highlighted that these activities are related to essential oil availability and interfere with the NF-κB pathway, causing a substantial decrease of NF-κB activation and, consequently, a significant reduction of some NF-κB target genes [109]. The same EO produced anti-proliferative and pro-apoptotic effects on HL-60 and its MDR variant HL-60R through the inhibition of NF-κB activation and the consequent reduction of expression, at the mRNA and protein levels, of some NF-κB targets [110].

*Alluaudia procera* essential oil has been tested in vitro on MDR models [111], in particular HL-60 and its MDR variant HL-60R to evaluate its antitumor abilities, and on Gram-negative *Escherichia coli* and Gram-positive *Staphylococcus aureus*, where it exhibited antibiotic resistance. This EO, in addition to terpenes such as precisely phytol, neophytadiene, and its isomers, contained five C16 macrocyclic lactones, of which Ambrettolide ((Z)-oxacycloheptadec-8-en-2-one) is the major component (18.1%). The latter could be attributed the biological activities that we have observed. In particular, *A. procera* EO induced a concentration-dependent reduction of the cancer cell viability of both cell lines and only an additive effect when EO was co-administrated with doxorubicin; for this, we supposed that essential oil or its major compounds are substrates of P-gp, as are many macrolides. Since the repeatedly administered doxorubicin was responsible for acquired resistance to chemotherapy due to its causing of an increase of P-gp expression via NF-κB, we hypothesized that C16 macrocyclic lactones could exert an antiproliferative effect by inhibiting the mammalian target of rapamycin (mTOR) and/or NF-κB like structural analogues [111].

Analog results obtained on AML cell lines with *C. juttae* EO have been described for the essential oil extracted from *Kalanchoe beharensis* [110]. Additionally, in this case, we observed an inhibition of cell growth and an induction of cell death, depending on the inhibition of the NF-κB pathway. This EO produced a significant decrease in many targets of NF-κB both at the mRNA and protein levels, like those of P-gp, IAPs, and Bcl-2, once again confirming the central role of NF-κB in the tumorigenesis process and, above all, in determining multi-drug resistance; all this makes it a potential pharmacological target for antitumor molecules [110].

In the case of the *Glandora rosmarinifolia* essential oil, we observed dose-dependent anti-proliferative activity—due, at least in part, to its pro-oxidant effects—on a panel of HCC cell lines (HA22T/VGH, HepG2, and Hep3B) and TNBC cell lines (SUM 149 and MDA-MB-231) [112]. Additionally, in this case, we assume that these activities depend by capacity to interfere on NF-κB activation, but this hypothesis remains to be verified.

#### 4.1.3. Triterpene Acids

Oleanolic acid (OA) and ursolic acid (UA) are triterpene acids endowed with biological properties, such as those of the anti-inflammatory, antimicrobial, and antidiabetic kinds [113,114,115].

Both these compounds are present in food vegetables to which they contribute chemo-preventive and antitumor properties [116,117,118]. Our interest in these molecules turned to their ability to interfere with NF-κB activation; we tested the cytotoxicity and inhibition capacity of the NF-κB activation of some OA and UA derivatives in HCC cell lines (HepG2, Hep3B, and HA22T/VGH). The chemical changes that were made had the purpose of increasing the lipophilia and/or oxidation state at C-3. Some analogs, in particular two epimeric compounds and a ketone, all derived from OA, showed a cytotoxic activity correlated with a strong NF-κB inhibition, once again demonstrating that this factor is a molecular target of those natural compounds with therapeutic actions that we call nutraceuticals [119].

### 4.2. Synthetic Compounds

#### 4.2.1. Dehydroxymethylepoxyquinomicin (DHMEQ): A Selective Inhibitor of NF-κB

Together with studies on substances of natural origin, our group also dealt with synthetic molecules with inhibitory activity on NF-κB. One of these is certainly dehydroxymethylepoxyquinomicin (DHMEQ), which inhibits the nuclear translocation of NF-κB. Currently, there is a great deal of scientific evidence on the efficacy of DHMEQ in different types of neoplastic and even inflammatory diseases, in vitro and in vivo [120,121,122,123,124]. We tested DHMEQ, for the first time, on HCC cell lines (HA22T/VGH, HepG2, and HuH-6), in which the molecule produced cytotoxic and pro-apoptotic effects in a proportional manner to levels of constitutively activated NF-κB in the three cell lines. In HA22T/VGH cells, in particular, we observed a synergy with cisplatin, both in the decrease cell growth and the induction of cell death effects. Moreover, in the same cell line, DHMEQ caused a dose-dependent reduction of activated factor levels and inhibited the activation induced by cisplatin. These effects turned into a decrease of expression of some NF-κB targets; specifically, DHMEQ inhibited IL-6 production, so we speculated that its antitumor activities may also have been due to an interference with a growth stimulatory autocrine loop based on IL-6 [125].

A strong reduction of NF-κB activation and the expression of some its targets (IAP1, Survivin, and XIAP) were observed on three TNBC cell lines (SUM 159, SUM 149, and MDA-MB-231) treated with DHMEQ [51].

#### 4.2.2. Proteasome Inhibitor

Examining the NF-κB pathway showed that any molecule able to interfere with its expression, activation, or degradation can be useful to downregulate this transcriptional factor, as well as with those of correlated factors. In this scenario, the proteasome inhibitors that block the degradation of IkBα by proteasome were studied as NF-κB inhibitors. The antitumor effects of NF-κB inhibitors like MG132 are now confirmed in many types of cancer [126,127,128].

We showed a synergistic cytotoxicity in TNBC cell lines when the cells were co-treated with MG132 and bisindolylmaleimide I, a PKC inhibitor with a high selectivity for PKCα-, β1-, β2-, γ-, δ, and ε-isozymes, as well as a very weak selectivity for PKCζ. We also observed a reduction of NF-κB activation that could only be attributed to a proteasome inhibitor. Presumably, the two molecules carried out a complex action on multiple pathways and not only on NF-κB [51].

#### 4.2.3. Epigenetic Agents

More recently, we focused on other factors related to NF-κB expression/activation, such as the Raf kinase inhibitor protein (RKIP). This behaves like a tumor suppressor in many types of cancer [129,130,131,132], as it inhibits the Raf-1/mitogen-activated protein kinase and, thus, the oncogenic activities related to this pathway [133]. There is a mutual influence between RKIP and NF-κB because RKIP affects the activation of nuclear factor, and, on the contrary, activated NF-κB is able to downregulate RKIP expression via Snail induction [134,135,136]. We have set ourselves the goal of understanding the molecular mechanisms responsible for the downregulation of RKIP in cancer cell to identify any molecules that simultaneously lead to the downregulation of NF-κB. Regarding epigenetic agents as demethylating agents or histone deacetylase (HDAC) inhibitor, their action on the NF-κB pathway is very controversial; in general, many scientific reports have shown how these agents cause the activation of the nuclear factor that may be inhibited by co-treatment with NF-κB inhibitors, e.g., vorinostat (HADACi) and bortezomib (proteasome inhibitor). For these reasons, setting aside the effects on NF-κB, we focused on the capacity of an HDAC inhibitor, trichostatin A (TSA), to enhance RKIP expression and inhibit invasion in a TBNC cell line, SUM 159. In these cells, co-treatment with TSA and DHMEQ produced synergistic effects on the inhibition of cell growth and the induction of apoptosis; overall, DHMEQ alone induced a strong increase in RKIP expression, both at the mRNA and protein levels [137].

#### 4.2.4. Silencing of MDA-9/Syntenin

Melanoma differentiation-associated gene-9/Syntenin (MDA-9/Syntenin), also called syndecan binding protein (SDCBP), is a protein related to NF-κB function because contributes to its activation through Src kinase activation.

For the first time, it was shown in melanoma cells as MDA-9/Syntenin/c-Src complexes that functionally cooperate with NF-κB to promote cellular growth and invasion [138]. Afterwards, an inverse relationship was highlighted between MDA-9 and RKIP in melanoma; MDA-9 downregulated RKIP at the transcriptional level, and, in contrast, ectopic RKIP expression in melanoma cells suppressed MDA-9-mediated signaling [139]. Consequently, by virtue of the mutual regulation between RKIP and NF-κB discussed above, there would be a loop among MDA-9/Syntenin, NF-κB, and RKIP that would constitute an important target for pharmacological approaches. In our study conducted on HCC cell lines (HA22T/VGH, Hep3B, and HepG2), we demonstrated that the silencing of MDA-9/Syntenin expression, obtained by a transient transfection with anti-MDA-9/Syntenin siRNA, downregulated NF-κB and the expression of some its targets while restoring the expression of RKIP, carrying out to strong reduction of MMP-2 dependent invasiveness [140]. 

A similar result was also achieved in TNBC cell models (SUM 159, SUM 149, and MDA-MB-231); siRNA anti-MDA-9/Syntenin produced a strong reduction of NF-κB activation and an increase of RKIP, with a reduction of cells’ invasion ability in all cell lines [51].

## 5. Other Scientific Evidence

It is clear that today we have a lot of data on NF-κB, and there is a lot of scientific evidence on molecules that have the ability to interfere with the pathway of this nuclear transcription factor. However, it is also true that many agents often present as highly variable depending on the context and type of tumor, as in the case of checkpoint inhibitors, which, despite some promising results, are not always effective because the action of anti-inflammatory stroma around cancer cells or the absence of infiltrating T cells can compromise the success of this approach. For this purpose, it appears necessary to understand the action of NF-κB on immune cells to prevent the increase of antitumor immunity due to the downregulation of NF-κB [141]. Many groups have suggested that the combination of immunotherapy with radio and chemotherapy might represent a promising therapeutic approach [142,143,144]. In Table 1, we report some clinical trials completed or in course regarding NF-κB as a drug target or as an important object of evaluation in the primary endpoints. Among pharmacological approaches, there are many types of anticancer drugs: natural ones (like curcumin and synthetic) that use the nuclear factor as the target and antitumor agents that interfere with pathways correlated with NF-κB. 

## 6. Conclusions

A huge amount of data strongly imply the involvement of transcription factor NF-κB in the onset and progression of a variety of tumors, highlighting its important role in modulating therapeutic efficacy, in particular on multidrug resistance cancers. Therefore, it is important to recognize the alterations in neoplastic cells that cause the overexpression and the hyperactivation of NF-κB, as well as to identify rational inhibitors of NF-κB or its upstream regulatory factors that can find applications as both autonomous therapies and adjuvants with existing or new therapies. In this regard, the findings of our and other groups support the possible use of natural multi-targeted agents like the polyphenols, triterpene acids, and essential oils characterized by a multiplicity of action, as well as synthetic drugs that behave like pure inhibitors of NF-κB or ones that are capable of interfering with pathways related to it; these can be used alone or in combination with conventional chemotherapeutic agents. Therefore, our future efforts will focus on the possibility of employing more effective analogues of natural compounds, as well as testing new synthetic compounds obtained by modifying already known agents such as DHMEQ.

## Figures and Tables

**Table 1:** Clinical trials completed or in course regarding NF-κB as drug target or as the important object of evaluation in the primary endpoints.

Identifier Number	Official Title	Date	Conclusions
**NCT00512798**	(Inhibition of NF-κB Signaling in Melanoma Therapy) A Phase I/II Clinical Trial of PS-341, a Proteasome Inhibitor, in Combination with an Extended Continuous Oral Schedule of Temozolomide in Patients with Advanced Refractory Solid Tumors with the Phase II Component Only in Patients with Melanoma	2003–2008	Terminated Due to Lack of Efficacy
**NCT03047837**	A Randomized, Phase II, Double-Blind, Placebo-controlled, Multicenter, 2x2 Factorial Design Biomarker Tertiary Prevention Trial of Low-dose Aspirin and Metformin in Stage I–III Colorectal Cancer Patients.	2017–2020	Not Terminated
**NCT01681368**	Phase II Open Label Non-Randomized Single Agent Study of the SMAC (Second Mitochondrial-Derived Activator of Caspases)-Mimetic Birinapant (TL32711; NSC 756502) in Relapsed Platinum Resistant or Refractory Epithelial Ovarian Cancer; Primary Peritoneal	2012–2014	Terminated Due to Lack of a Clinical Benefit
**NCT04208334**	A Double-blind, Placebo-Controlled Randomized Trial Phase II Evaluating the Effect of Curcumin for Treatment of Cancer Anorexia–Cachexia Syndrome in Patients with Stage III–IV of Head and Neck Cancer	2020–2020	Not Terminated
**NCT04234022**	Zn-DDC to Target Hypoxia-NF-ĸB–CSCs Pathway and Improve the Treatment Outcomes of Hematological Malignancies—A Translation Bench Study	2020–2023	Not Terminated (Not Yet Recruiting)
**NCT01740323**	Meriva for Treatment-Induced Inflammation and Fatigue in Women with Breast Cancer	2015–2018	Completed (the Study Only Highlighted a Slight Advantage by Curcumin Treatment, Compared to Placebo, to Reduce Symptoms of Fatigue Associated with Excessive NF-ĸB Activity in BRCA Patients)
**NCT00899353**	Inhibition of NF-ĸB for Prevention of Disease Progression in Indolent B Cell Malignancies	2008–2012	Terminated (Original Principal Investigator Left the Institution.)
**NCT02765854**	Phase II Trial of Ixazomib and Dexamethasone Versus Ixazomib, Dexamethasone, and Lenalidomide, Randomized with NF-kB2 Rearrangement (Proteasome Inhibitor NF-kB2 Rearrangement Driven Trial: PINR)	2016–2021	Not Terminated
**NCT01132911**	A Phase I Study of Vorinostat and Bortezomib in Children with Refractory of Recurrent Solid Tumors, Including CNS Tumors and Lymphomas	2010–2011	Completed (No Study Results Posted on ClinicalTrials.gov)
**NCT02144675**	A Randomized Phase II Study of NF-κB Inhibition During Induction Chemotherapy for Patients with Acute Myelogenous Leukemia	2009–2016	Completed (It Was Not Reported if the Treatment with Choline Magnesium Trisalicylate and Chemotherapy Produced a Modulation of NF-ĸB Transcriptional Targets and/or Drug Efflux in at Least 50% of Patients)
**NCT03978624**	Window of Opportunity Platform Study to Define Immunogenomic Changes with Pembrolizumab Alone and in Rational Combinations in Muscle-Invasive Bladder Cancer	2019–2021	Not Terminated
**NCT00280761**	A Biologic Study of Global Gene Expression, NF-Kappa B and p53 in Adenocarcinoma of the Rectum	2003–2021	Not Terminated
**NCT00541359**	A Phase I Trial of PS-341 in Combination with Topotecan in Advanced Solid Tumor Malignancies	2004–2011	Completed (No Study Results Posted on ClinicalTrials.gov)
**NCT00305734**	Phase II Trial of PS-341 (Bortezomib, NSC-681239) Followed by the Addition of Gemcitabine at Progression in Recurrent or Metastatic Nasopharyngeal Carcinoma	2006–2007	Completed (No Study Results Posted on ClinicalTrials.gov)
**NCT00745134**	A Randomized Double Blinded Study of Curcumin with Pre-Operative Capecitabine and Radiation Therapy Followed by Surgery for Rectal Cancer	2008–2021	Not Terminated
**NCT03382340**	A Phase 1/2a Open-Label, Dose-Escalation/Dose-Expansion Safety, Tolerability and Pharmacokinetic Study of IMX-110 (a Nanoparticle Encapsulating a Stat3/NF-kB/Poly-Tyrosine Kinase Inhibitor and Low-Dose Doxorubicin) in Patients with Advanced Solid Tumors	2018–2021	Not terminated
**NCT00113841**	Pilot Study of Curcumin (Diferuloylmethane Derivative) with or without Bioperine in Patients with Multiple Myeloma	2004–2009	Completed (Curcumin and bioperine compared to Curcumin Alone Produced a Higher Reduction of NF-ĸB and Its Related Gene Expression in the Multiple Myeloma cells).
**NCT00305747**	Phase I Study of BioResponse-Dim in Non-Metastatic, Hormone-Refractory Prostate Cancer Patients with Rising Serum PSA	2005–2010	Completed (No Study Results Posted on ClinicalTrials.gov)
**NCT01269203**	A Phase II Randomized Study of the Efficacy of Curcumin for Reducing Symptoms During Maintenance Therapy in Multiple Myeloma Patients	2012–2015	Withdrawn
**NCT00156299**	A Pilot Study of NF-kB Inhibition During Induction Chemotherapy for Patients with Acute Myelogenous Leukemia (AML)	2003–2008	Terminated (Replaced by Another Study)
**NCT02944578**	Biomolecular Effects of Topical Curcumin in HSIL Cervical Neoplasia	2017–2021	Not Terminated

## References

[1] Gilmore T.D., Herscovitch M. (2006). Inhibitors of NF-kappaB signaling: 785 and counting. Oncogene.

[2] Hayden M.S., Ghosh S. (2004). Signaling to NF-kappaB. Genes Dev..

[3] Aggarwal B.B. (2004). Nuclear factor-kappaB: The enemy within. Cancer Cell.

[4] Gilmore T.D., Kalaitzidis D., Liang M.C., Starczynowsk D.T. (2004). The c-Rel transcription factor and B-cell proliferation: A deal with the devil. Oncogene.

[5] Braun T., Carvalho G., Coquelle A., Vozenin M.C., Lepelley P., Hirsch F., Kiladjian J.J., Ribrag V., Fenaux P., Kroemer G. (2006). NF-kappaB constitutes a potential therapeutic target in high-risk myelodysplastic syndrome. Blood.

[6] Annunziata C.M., Stavnes H.T., Kleinberg L., Berner A., Hernandez L.F., Birrer M.J., Steinberg S.M., Davidson B., Kohn E.C. (2010). Nuclear factor κB transcription factors are coexpressed and convey a poor outcome in ovarian cancer. Cancer.

[7] Codony-Servat J., Marín-Aguilera M., Visa L., García-Albéniz X., Pineda E., Fernández P.L., Filella X., Gascón P., Mellado B. (2013). Nuclear factor-kappa B and interleukin-6 related docetaxel resistance in castration-resistant prostate cancer. Prostate.

[8] Long Y.M., Ye S., Rong J., Xie W.R. (2008). Nuclear factor kappa B: A marker of chemotherapy for human stage IV gastric carcinoma. World J. Gastroenterol..

[9] Plewka D., Plewka A., Miskiewicz A., Morek M., Bogunia E. (2018). Nuclear factor-kappa B as potential therapeutic target in human colon cancer. J. Cancer Res. Ther..

[10] Yang Y.M., Kim S.Y., Seki E. (2019). Inflammation and Liver Cancer: Molecular Mechanisms and Therapeutic Targets, Inflammation and Liver Cancer. Semin. Liver Dis..

[11] Prajoko Y.W., Aryandono T. (2014). Expression of nuclear factor kappa B (NF-kB) as a predictor of poor pathologic response to chemotherapy in patients with locally advanced breast cancer. Asian Pac. J. Cancer Prev..

[12] Ma C., Zu X., Liu K., Bode A.M., Dong Z., Liu Z., Kim D.J. (2019). Knockdown of Pyruvate Kinase M Inhibits Cell Growth and Migration by Reducing NF-kB Activity in Triple-Negative Breast Cancer Cells. Mol Cells.

[13] Basseres D.S., Baldwin A.S. (2006). Nuclear factor-kappaB and inhibitor of kappaB kinase pathways in oncogenic initiation and progression. Oncogene.

[14] Greenman C., Stephens P., Smith R., Dalgliesh G.L., Hunter C., Bignell G., Davies H., Teague J., Butler A., Stevens C. (2007). Patterns of somatic mutation in human cancer genomes. Nature.

[15] Xia Y., Shen S., Verma I.M. (2014). NF-κB, an active player in human cancers. Cancer Immunol. Res..

[16] Bentires-Alj M., Barbu V., Fillet M., Chariot A., Relic B., Jacobs N., Gielen J., Merville M.P., Bours V. (2003). NF-kappaB transcription factor induces drug resistance through MDR1 expression in cancer cells. Oncogene.

[17] Colotta F., Allavena P., Sica A., Garlanda C., Mantovani A. (2009). Cancer-related inflammation, the seventh hallmark of cancer: Links to genetic instability. Carcinogenesis.

[18] Elinav E., Nowarski R., Thaiss C.A., Hu B., Jin C., Flavell R.A. (2013). Inflammation-induced cancer: Crosstalk between tumours, immune cells and microorganisms. Nat. Rev. Cancer.

[19] Hanahan D., Weinberg R.A. (2000). The hallmarks of cancer. Cell.

[20] Burstein E., Duckett C.S. (2003). Dying for NF-kappaB? Control of cell death by transcriptional regulation of the apoptotic machinery. Curr. Opin. Cell Biol..

[21] Kucharczak J., Simmons M.J., Fan Y., Gélinas C. (2003). To be, or not to be: NF-kappaB is the answer--role of Rel/NF-kappaB in the regulation of apoptosis. Oncogene.

[22] Huang S., Pettaway C.A., Uehara H., Bucana C.D., Fidler I.J. (2001). Blockade of NF-kappaB activity in human prostate cancer cells is associated with suppression of angiogenesis, invasion, and metastasis. Oncogene.

[23] Karin M., Cao Y., Greten F.R., Li Z.W. (2002). NF-kappaB in cancer: From innocent bystander to major culprit. Nat. Rev. Cancer.

[24] Huber M.A., Azoitei N., Baumann B., Grünert S., Sommer A., Pehamberger H., Kraut N., Beug H., Wirth T. (2004). NF-kappaB is essential for epithelial-mesenchymal transition and metastasis in a model of breast cancer progression. J. Clin. Investig..

[25] Huber M.A., Beug H., Wirth T. (2004). Epithelial-mesenchymal transition: NF-kappaB takes center stage. Cell Cycle.

[26] Boukerche H., Su Z.Z., Emdad L., Sarkar D., Fisher P.B. (2007). mda-9/Syntenin regulates the metastatic phenotype in human melanoma cells by activating nuclear factor-kappaB. Cancer Res..

[27] Talukdar S., Das S.K., Pradhan A.K., Emdad L., Windle J.J., Sarkar D., Fisher P.B. (2019). MDA-9/Syntenin (SDCBP) Is a Critical Regulator of Chemoresistance, Survival and Stemness in Prostate Cancer Stem Cells. Cancers (Basel).

[28] Bhoopathi P., Pradhan A.K., Bacolod M.D., Emdad L., Sarkar D., Das S.K., Fisher P.B. (2019). Regulation of neuroblastoma migration, invasion, and in vivo metastasis by genetic and pharmacological manipulation of MDA-9/Syntenin. Oncogene.

[29] Das S.K., Sarkar D., Cavenee W.K., Emdad L., Fisher P.B. (2019). Rethinking Glioblastoma Therapy: MDA-9/Syntenin Targeted Small Molecule. ACS Chem. Neurosci..

[30] Das S.K., Maji S., Wechman S.L., Bhoopathi P., Pradhan A.K., Talukdar S., Sarkar D., Landry J., Guo C., Wang X.Y. (2020). MDA-9/Syntenin (SDCBP): Novel gene and therapeutic target for cancer metastasis. Pharmacol. Res..

[31] Bednarski B.K., Ding X., Coombe K., Baldwin A.S., Kim H.J. (2008). Active roles for inhibitory kappaB kinases alpha and beta in nuclear factor-kappaB-mediated chemoresistance to doxorubicin. Mol. Cancer Ther..

[32] Wang Z., Zhang L., Ni Z., Sun J., Gao H., Cheng Z., Xu J., Yin P. (2015). Resveratrol induces AMPK-dependent MDR1 inhibition in colorectal cancer HCT116/L-OHP cells by preventing activation of NF-κB signaling and suppressing cAMP-responsive element transcriptional activity. Tumor Biol..

[33] Suttana W., Mankhetkorn S., Poompimon W., Palagani A., Zhokhov S., Gerlo S., Haegeman G., Berghe W.V. (2010). Differential chemosensitization of P-glycoprotein overexpressing K562/Adr cells by withaferin A and Siamois polyphenols. Mol. Cancer.

[34] Xia Y.Z., Ni K., Guo C., Zhang C., Geng Y.D., Wang Z.D., Yang L., Kong L.Y. (2015). Alopecurone B reverses doxorubicin-resistant human osteosarcoma cell line by inhibiting P-glycoprotein and NF-kappa B signaling. Phytomedicine.

[35] Filipits M. (2004). Mechanisms of cancer: Multidrug resistance. Drug Discov. Today Dis. Mech..

[36] Saraswathy M., Gong S. (2013). Different strategies to overcome multidrug resistance in cancer. Biotechnol. Adv..

[37] Advani P., Moreno-Aspitia A. (2014). Current strategies for the prevention of breast cancer. Breast Cancer.

[38] Lebert J.M., Lester R., Powell E., Seal M., McCarthy J. (2018). Advances in the systemic treatment of triple-negative breast cancer. Curr. Oncol..

[39] Schmadeka R., Harmon B.E., Singh M. (2014). Triple-negative breast carcinoma: Current and emerging concepts. Am. J. Clin. Pathol..

[40] Foulkes W.D., Smith I.E., Reis-Filho J.S. (2010). Triple-negative breast cancer. N. Engl. J. Med..

[41] Russnes H.G., Navin N., Hicks J., Borresen-Dale A.L. (2011). Insight into the heterogeneity of breast cancer through next-generation sequencing. J. Clin. Investig..

[42] Sethi S., Sarkar F.H., Ahmed Q., Bandyopadhyay S., Nahleh Z.A., Semaan A., Sakr W., Munkarah A., Ali-Fehmi R. (2011). Molecular markers of epithelial-to-mesenchymal transition are associated with tumor aggressiveness in breast carcinoma. Transl. Oncol..

[43] Piao H.L., Yuan Y., Wang M., Sun Y., Liang H., Ma L. (2014). α-catenin acts as a tumour suppressor in E-cadherin-negative basal-like breast cancer by inhibiting NF-κB signalling. Nat. Cell Biol..

[44] Barbie T.U., Alexe G., Aref A.R., Li S., Zhu Z., Zhang X., Imamura Y., Thai T.C., Huang Y., Bowden M. (2014). Targeting an IKBKE cytokine network impairs triple-negative breast cancer growth. J. Clin. Investig..

[45] Yamaguchi N., Ito T., Azuma S., Ito E., Honma R., Yanagisawa Y., Nishikawa A., Kawamura M., Imai J., Watanabe S. (2009). Constitutive activation of nuclear factor-kappaB is preferentially involved in the proliferation of basal-like subtype breast cancer cell lines. Cancer Sci..

[46] Ossovskaya V., Wang Y., Budoff A., Xu Q., Lituev A., Potapova O., Vansant G., Monforte J., Daraselia N. (2011). Exploring molecular pathways of triple-negative breast cancer. Genes Cancer.

[47] Cogswell P.C., Guttridge D.C., Funkhouser W.K., Baldwin A.S. (2000). Selective activation of NF-kappa B subunits in human breast cancer: Potential roles for NF-kappa B2/p52 and for Bcl-3. Oncogene.

[48] Lee S.T., Li Z., Wu Z., Aau M., Guan P., Karuturi R.K., Liou Y.C., Yu Q. (2011). Context-specific regulation of NF-κB target gene expression by EZH2 in breast cancers. Mol. Cell.

[49] D’Alessandro N., Poma P., Labbozzetta M., Vivona N., Notarbartolo M. (2014). Mechanisms of Raf-1 Kinase Inhibitor Protein Dysregulation in Triple-Negative Breast Cancers and Identification of Possible Novel Therapeutic Approaches for These Tumors. Forum Immunopathol. Dis. Ther..

[50] Poma P., Notarbartolo M., Labbozzetta M., Maurici A., Carina V., Alaimo A., Rizzi M., Simoni D., D’Alessandro N. (2007). The antitumor activities of curcumin and of its isoxazole analogue are not affected by multiple gene expression changes in an MDR model of the MCF-7 breast cancer cell line: Analysis of the possible molecular basis. Int. J. Mol. Med..

[51] Poma P., Labbozzetta M., D’Alessandro N., Notarbartolo M. (2017). NF-κB Is a Potential Molecular Drug Target in Triple-Negative Breast Cancers. OMICS.

[52] Chavez K.J., Garimella S.V., Lipkowitz S. (2010). Triple Negative Breast Cancer Cell Lines: One Tool in the Search for Better Treatment of Triple Negative Breast Cancer. Breast Dis..

[53] Yadav B.S., Sharma S.C., Chanana P., Jhamb S. (2014). Systemic treatment strategies for triple-negative breast cancer. World J. Clin. Oncol..

[54] Abramson V.G., Lehmann B.D., Ballinger T.J., Pietenpol J.A. (2015). Subtyping of triple-negative breast cancer: Implications for therapy. Cancer.

[55] De Graffenried L.A., Chandrasekar B., Friedrichs W.E., Donzis E., Silva J., Hidalgo M., Freeman J.W., Weiss G.R. (2004). NF-kB inhibition markedly enhances sensitivity of resistant breast cancer tumor cells to tamoxifen. Ann. Oncol..

[56] Llovet J.M., Zucman-Rossi J., Pikarsky E., Sangro B., Schwartz M., Sherman M., Gores G. (2016). Hepatocellular carcinoma. Nat. Rev. Dis. Primers.

[57] Haybaeck J., Zeller N., Wolf M.J., Weber A., Wagner U., Kurrer M.O., Bremer J., Iezzi G., Graf R., Clavien P.A. (2009). A lymphotoxin-driven pathway to hepatocellular carcinoma. Cancer Cell.

[58] Liu P., Kimmoun E., Legrand A., Sauvanet A., Degott C., Lardeux B., Bernuau D. (2002). Activation of NF-kappaB, AP-1 and STAT transcription factors is a frequent and early event in human hepatocellular carcinomas. J. Hepatol..

[59] Arsura M., Cavin L.G. (2005). Nuclear factor-kappaB and liver carcinogenesis. Cancer Lett..

[60] Vainer G.W., Pikarsky E., Ben-Neriah Y. (2008). Contradictory functions of NF-kB in liver physiology and cancer. Cancer Lett..

[61] Pikarsky E., Porat R.M., Stein I., Abramovitch R., Amit S., Kasem S., Gutkovich-Pyest E., Urieli-Shoval S., Galun E., Ben-Neriah Y. (2004). NF-kappaB functions as a tumour promoter in inflammation-associated cancer. Nature.

[62] Luedde T., Beraza N., Kotsikoris V., van Loo G., Nenci A., De Vos R., Roskams T., Trautwein C., Pasparakis M. (2007). Deletion of NEMO/IKKgamma in liver parenchymal cells causes steatohepatitis and hepatocellular carcinoma. Cancer Cell.

[63] Sunami Y., Ringelhan M., Kokai E., Lu M., O’Connor T., Lorentzen A., Weber A., Rodewald A.K., Mullhaupt B., Terracciano L. (2016). Canonical NF-kappaB signaling in hepatocytes acts as a tumor-suppressor in hepatitis B virus surface antigen-driven hepatocellular carcinoma by controlling the unfolded protein response. Hepatology.

[64] Chiao P.J., Na R., Niu J., Sclabas G.M., Dong Q., Curley S.A. (2002). Role of Rel/NF-kappaB transcription factors in apoptosis of human hepatocellular carcinoma cells. Cancer.

[65] Robey R.W., Pluchino K.M., Hall M.D., Fojo A.T., Bates S.E., Gottesman M.M. (2018). Revisiting the role of ABC transporters in multidrug-resistant cancer. Nat. Rev. Canc..

[66] Notarbartolo M., Poma P., Perri D., Dusonchet L., Cervello M., D’Alessandro N. (2005). Antitumor effects of curcumin, alone or in combination with cisplatin or doxorubicin, on human hepatic cancer cells. Analysis of their possible relationship to changes in NF-kB activation levels and in IAP gene expression. Cancer Lett..

[67] Al-Bahar S., Adriana Z., Pandita R. (2008). Acute myeloid leukemia as a genetic disease. Gulf J. Oncol..

[68] Zhou J., Ching Y.Q., Chng W.J. (2015). Aberrant nuclear factor-kappa B activity in acute myeloid leukemia: From molecular pathogenesis to therapeutic target. Oncotarget.

[69] Grandage V.L., Gale R.E., Linch D.C., Khwaja A. (2005). PI3-kinase/Akt is constitutively active in primary acute myeloid leukaemia cells and regulates survival and chemoresistance via NFkB, MAPkinase and p53 pathways. Leukemia.

[70] Pan L., Li Y., Zhang H.Y., Zheng Y., Liu X.L., Hu Z., Wang Y., Wang J., Cai Y.H., Liu Q. (2017). DHX15 is associated with poor prognosis in acute myeloid leukemia (AML) and regulates cell apoptosis via the NF-kB signaling pathway. Oncotarget.

[71] Cilloni D., Martinelli G., Messa F., Baccarani M., Saglio G. (2007). Nuclear factor k B as a target for new drug development in myeloid malignancies. Haematologica.

[72] Terzic J., Grivennikov S., Karin E., Karin M. (2010). Inflammation and colon cancer. Gastroenterology.

[73] Brandao M.M., Soares E., Salles T.S., Saad S.T. (2001). Expression of inducible nitric oxide synthase is increased in acute myeloid leukaemia. Acta Haematol..

[74] Hjortso M.D., Andersen M.H. (2014). The expression, function and targeting of haem oxygenase-1 in cancer. Curr. Cancer Drug Targets.

[75] Rushworth S.A., MacEwan D.J. (2008). HO-1 underlies resistance of AML cells to TNF-induced apoptosis. Blood.

[76] Baumgartner B., Weber M., Quirling M., Fischer C., Page S., Adam M., Von Schilling C., Waterhouse C., Schmid C., Neumeier D. (2002). Increased IkappaB kinase activity is associated with activated NF-kappaB in acute myeloid blasts. Leukemia.

[77] Notarbartolo M., Cervello M., Dusonchet L., Cusimano A., D’Alessandro N. (2002). Resistance to diverse apoptotic triggers in multidrug resistant HL60 cells and its possible relationship to the expression of P-glycoprotein, Fas and of the novel anti-apoptosis factors IAP (inhibitory of apoptosis proteins). Cancer Lett..

[78] Notarbartolo M., Cervello M., Poma P., Dusonchet L., Meli M., D’Alessandro N. (2004). Expression of the IAPs in multidrug resistant tumor cells. Oncol. Rep..

[79] Tamm I., Kornblau S.M., Segall H., Krajewski S., Welsh K., Kitada S., Scudiero D.A., Tudor G., Qui Y.H., Monks A. (2000). Expression and prognostic significance of IAP-family genes in human cancers and myeloid leukemias. Clin. Cancer Res..

[80] Hrdinka M., Yabal M. (2019). Inhibitor of apoptosis proteins in human health and disease. Genes Immun..

[81] Darwish N.H.E., Sudha T., Godugu K., Bharali D.J., Elbaz O., El-Ghaffar H.A.A., Azmy E., Anber N., Mousa S.A. (2019). Novel Targeted Nano-Parthenolide Molecule against NF-kB in Acute Myeloid Leukemia. Molecule..

[82] Stone A., Zukerman T., Flaishon L., Yakar R.B., Rowe J.M. (2019). Efficacy outcomes in the treatment of older or medically unfit patients with acute myeloid leukaemia: A systematic review and meta-analysis. Leuk. Res..

[83] Manson M.M. (2005). Inhibition of survival signalling by dietary polyphenols and indole-3-carbinol. Eur. J. Cancer.

[84] Pojero F., Poma P., Spanò V., Montalbano A., Barraja P., Notarbartolo M. (2019). Targeting multiple myeloma with natural polyphenols. Eur. J. Med. Chem..

[85] Shishodia S., Potdar P., Gairola C.G., Aggarwal B.B. (2003). Curcumin (diferuloylmethane) down-regulates cigarette smoke-induced NF-kappaB activation through inhibition of IkappaBalpha kinase in human lung epithelial cells: Correlation with suppression of COX-2, MMP-9 and cyclin D1. Carcinogenesis.

[86] Aggarwal B.B., Kumar A., Bharti A.C. (2003). Anticancer potential of curcumin: Preclinical and clinical studies. Anticancer Res..

[87] Labbozzetta M., Notarbartolo M., Poma P., Maurici A., Inguglia L., Marchetti P., Rizzi M., Baruchello R., Simoni D., D’Alessandro N. (2009). Curcumin as a possible lead compound against hormone-indipendent multidrug resistant breast cancer. Ann. N. Y. Acad. Sci..

[88] Simon A., Allais D.P., Duroux J.L., Basly J.P., Durand-Fontanier S., Delage C. (1998). Inhibitory effect of curcuminoids on MCF-7 cell proliferation and structure-activity relationships. Cancer Lett..

[89] Robinson T.P., Ehlers T., Hubbard R.B., Bai X., Arbiser J.L., Goldsmith D.J., Bowen J.P. (2003). Design, synthesis, and biological evaluation of angiogenesis inhibitors: Aromatic enone and dienone analogues of curcumin. Bioorg. Med. Chem. Lett..

[90] Mosley C.A., Liotta D.C., Snyder J.P. (2007). Highly active anticancer curcumin analogues. Adv. Exp. Med. Biol..

[91] Simoni D., Rizzi M., Rondanin R., Baruchello R., Marchetti P., Invidiata F.P., Labbozzetta M., Poma P., Carina V., Notarbartolo M. (2008). Antitumor effects of curcumin and structurally b-diketone modified analogs on multidrug resistant cancer cells. Bioorg. Med. Chem. Lett..

[92] De Vreese R., Grootaert C., D’hoore S., Theppawong A., Van Damme S., Van Bogaert M., Van Camp J., D’hooghe M. (2016). Synthesis of novel curcuminoids accommodating a central β-enaminone motif and their impact on cell growth and oxidative stress. Eur. J. Med. Chem..

[93] Weber W.M., Hunsaker L.A., Abcouwer S.F., Deck L.M., Vander Jagt D.L. (2005). Anti-oxidant activities of curcumin and related enones. Bioorg. Med. Chem..

[94] Bonaccorsi P.M., Labbozzetta M., Barattucci A., Salerno T.M.G., Poma P., Notarbartolo M. (2019). Synthesis of Curcumin Derivatives and Analysis of Their Antitumor Effects in Triple Negative Breast Cancer (TNBC) Cell Lines. Pharmaceuticals.

[95] Limtrakul P. (2007). Curcumin as a chemosensitizer. Adv. Exp. Med. Biol..

[96] Kurien B.T., Singh A., Matsumoto H., Scofield R.H. (2007). Improving the solubility and pharmacological efficacy of curcumin by heat treatment. Assay Drug Dev. Technol..

[97] Wang Y.J., Pan M.H., Cheng A.L., Lin L.I., Ho Y.S., Hsieh C.Y., Lin J.K. (1997). Stability of curcumin in buffer solutions and characterization of itsdegradation products. J. Pharm. Biomed. Anal..

[98] Anand P., Kunnumakkara A.B., Newman R.A., Aggarwal B.B. (2007). Bioavailability of curcumin: Problems and promises. Mol. Pharm..

[99] Amano C., Minematsu H., Fujita K., Iwashita S., Adachi M., Igarashi K., Hinuma S. (2015). Nanoparticles containing curcumin useful for suppressing macrophages in vivo in mice. PLoS ONE.

[100] Chow E.K.H., Ho D. (2013). Cancer nanomedicine: From drug delivery to imaging. Sci. Transl. Med..

[101] Nicolas J., Mura S., Brambilla D., Mackiewicz N., Couvreur P. (2013). Design, functionalization strategies and biomedical applications of targeted biodegradable/biocompatible polymer-based nanocarriers for drug delivery. Chem. Soc. Rev..

[102] Massaro M., Amorati R., Cavallaro G., Guernelli S., Lazzara G., Milioto S., Noto R., Poma P., Riela S. (2016). Direct chemical grafted curcumin on halloysite nanotubes as dual-responsive prodrug for pharmacological applications. Colloids Surf. B Biointerfaces.

[103] Massaro M., Poma P., Colletti C.G., Barattucci A., Bonaccorsi P.M., Lazzara G., Nicotra G., Parisi F., Salerno T.M.G., Spinella C. (2020). Chemical and biological evaluation of cross-linked halloysite-curcumin derivatives. Appl. Clay Sci..

[104] Teklehaymanot T., Giday M. (2007). Ethnobotanical study of medicinal plants used by people in Zegie Peninsula, Northwestern Ethiopia. J. Ethnobiol. Ethnomed..

[105] Crellin J.K., Philpott J., Bass A.L.T. (1990). Herbal Medicine Past and Present: A Reference Guide to Medicinal Plants.

[106] Fernandes F., Andrade P.B., Ferreres F., Gil-Izquierdo A., Sousa-Pinto I., Valentão P. (2017). The chemical composition on fingerprint of Glandora diffusa and its biological properties. Arabian J Chem..

[107] Bakkali F., Averbeck S., Averbeck D., Idaomar M. (2008). Biological effects of essential oils—A review. Food Chem. Toxicol..

[108] Milad R., El-Ahmady S., Singab A.N. (2014). Genus Kalanchoe (Crassulaceae): A review of its ethnomedicinal, botanical, chemical and pharmacological properties. Eur. J. Med. Plants.

[109] Zito P., Labbozzetta M., Notarbartolo M., Sajeva M., Poma P. (2019). Essential oil of Cyphostemma juttae (Vitaceae): Chemical composition and antitumor mechanism in triple negative breast cancer cells. PLoS ONE.

[110] Poma P., Labbozzetta M., McCubrey J.A., Ramarosandratana A.V., Sajeva M., Zito P., Notarbartolo M. (2019). Antitumor Mechanism of the Essential Oils from Two Succulent Plants in Multidrug Resistance Leukemia Cell. Pharmaceuticals.

[111] Poma P., Labbozzetta M., Zito P., Alduina R., Ramarosandratana A.V., Bruno M., Rosselli S., Sajeva M., Notarbartolo M. (2019). Essential Oil Composition of Alluaudia procera and in Vitro Biological Activity on Two Drug-Resistant Models. Molecules.

[112] Poma P., Labbozzetta M., Notarbartolo M., Bruno M., Maggio A., Rosselli S., Sajeva M., Zito P. (2018). Chemical composition, in vitro antitumor and pro-oxidant activities of Glandora rosmarinifolia (Boraginaceae) essential oil. PLoS ONE.

[113] Paszel-Jaworska A., Romaniuk A., Rybczynska M. (2014). Molecular mechanisms of biological activity of oleanolic acid—A source of inspiration for a new drugs design. Mini-Rev. Org. Chem..

[114] Chen H., Gao Y., Wang A., Zhou X., Zheng Y., Zhou J. (2015). Evolution in medicinal chemistry of ursolic acid derivatives as anticancer agents. Eur. J. Med. Chem..

[115] Meng Y.Q., Liu D., Cai L.L., Chen H., Cao B., Wang Y.Z. (2009). The synthesis of ursolic acid derivatives with cytotoxic activity and the investigation of their preliminary mechanism of action. Biorg. Med. Chem..

[116] Neto C.C. (2007). Cranberry and blueberry: Evidence for protective effects against cancer and vascular diseases. Mol. Nutr. Food Res..

[117] Svarcova I., Heinrich J., Valentova K. (2007). Berry fruits as a source of biologically active compounds: The case of Lonicera caerulea. Biomed. Pap. Med. Fac. Univ. Palacky Olomouc Czech. Repub..

[118] Sanchez-Quesada C., Lopez-Biedma A., Warleta F., Campos M., Beltran G., Gaforio J.J. (2013). Bioactive properties of the main Triterpenes found in Olives, Virgin Olive Oil, and Leaves of Olea europaea. J. Agric. Food Chem..

[119] Fontana G., Bruno M., Notarbartolo M., Labbozzetta M., Poma P., Spinella A., Rosselli S. (2019). Cytotoxicity of oleanolic and ursolic acid derivatives toward hepatocellular carcinoma and evaluation of NF-κB involvement. Bioorg. Chem..

[120] Katsman A., Umezawa K., Bonavida B. (2009). Chemosensitization and immunosensitization of resistant cancer cells to apoptosis and inhibition of metastasis by the specific NF-kappaB inhibitor DHMEQ. Curr. Pharm. De.s.

[121] Ukaji T., Umezawa K. (2014). Novel approaches to target NF-κB and other signaling pathways in cancer stem cells. Adv. Biol. Regul..

[122] Lin Y., Ukaji T., Koide N., Umezawa K. (2018). Inhibition of Late and Early Phases of Cancer Metastasis by the NF-κB Inhibitor DHMEQ Derived from Microbial Bioactive Metabolite Epoxyquinomicin: A Review. Int. J. Mol. Sci..

[123] Jiang X., He H., Xie Z., Wen H., Li X., Li X., Ma J., Umezawa K., Zhang Y. (2019). Dehydroxymethylepoxyquinomicin suppresses atopic dermatitis-like lesions in a stratum corneum-removed murine model through NF-κB inhibition. Immunopharmacol. Immunotoxicol..

[124] Ando Y., Keino H., Kudo A., Hirakata A., Okada A.A., Umezawa K. (2020). Anti-Inflammatory Effect of Dehydroxymethylepoxyquinomicin, a Nuclear factor-κB Inhibitor, on Endotoxin-Induced Uveitis in Rats In vivo and In vitro. Ocul. Immunol. Inflamm..

[125] Poma P., Notarbartolo M., Labbozzetta M., Sanguedolce R., Alaimo A., Carina V., Maurici A., Cusimano A., Cervello M., D’Alessandro N. (2006). Antitumor effects of the novel NF-kappaB inhibitor dehydroxymethyl-epoxyquinomicin on human hepatic cancer cells: Analysis of synergy with cisplatin and of possible correlation with inhibition of pro-survival genes and IL-6 production. Int. J. Oncol..

[126] Qureshi A.A., Zuvanich E.G., Khan D.A., Mushtaq S., Silswal N., Qureshi N. (2018). Proteasome inhibitors modulate anticancer and anti-proliferative properties via NF-kB signaling, and ubiquitin-proteasome pathways in cancer cell lines of different organs. Lipids Health Dis..

[127] Narayanan S., Cai C.Y., Assaraf Y.G., Guo H.Q., Cui Q., Wei L., Huang J.J., Ashby C.R., Chen Z.S. (2019). Targeting the ubiquitin-proteasome pathway to overcome anti-cancer drug resistance. Drug Resist. Updat..

[128] Shalem-Cohavi N., Beery E., Nordenberg J., Rozovski U., Raanani P., Lahav M., Uziel O. (2019). The Effects of Proteasome Inhibitors on Telomerase Activity and Regulation in Multiple Myeloma Cells. Int J Mol Sci.

[129] Minn A.J., Bevilacqua E., Yun J., Rosner M.R. (2012). Identification of novel metastasis suppressor signaling pathways for breast cancer. Cell Cycle.

[130] Yeung K., Seitz T., Li S., Janosch P., McFerran B., Kaiser C., Fee F., Katsanakis K.D., Rose D.W., Mischak H. (1999). Suppression of Raf-1 kinase activity and MAP kinase signalling by RKIP. Nature.

[131] Schuierer M.M., Bataille F., Weiss T.S., Hellerbrand C., Bosserhoff A.K. (2006). Raf kinase inhibitor protein is downregulated in hepatocellular carcinoma. Oncol. Rep..

[132] Zaravinos A., Bonavida B., Chatzaki E., Baritaki S. (2018). RKIP: A Key Regulator in Tumor Metastasis Initiation and Resistance to Apoptosis: Therapeutic Targeting and Impact. Cancers (Basel).

[133] Odabaei G., Chatterjee D., Jazirehi A.R., Goodglick L., Yeung K., Bonavida B. (2004). Raf-1 kinase inhibitor protein: Structure, function, regulation of cell signaling and pivotal role in apoptosis. Adv. Cancer Res..

[134] Tang H., Park S., Sun S.C., Trumbly R., Ren G., Tsung E., Yeung K.C. (2010). RKIP inhibits NF-kappaB in cancer cells by regulating upstream signaling components of the IkappaB kinase complex. FEBS Lett..

[135] Wu K., Bonavida B. (2009). The activated NF-kappaB-Snail-RKIP circuitry in cancer regulates both the metastatic cascade and resistance to apoptosis by cytotoxic drugs. Crit. Rev. Immunol..

[136] Jing S.H., Gao X., Yu B., Qiao H. (2017). Raf Kinase Inhibitor Protein (RKIP) Inhibits Tumor Necrosis Factor-α (TNF-α) Induced Adhesion Molecules Expression in Vascular Smooth Muscle Bells by Suppressing (Nuclear Transcription Factor-κB (NF-kappaB) Pathway. Med. Sci. Monit..

[137] Labbozzetta M., Poma P., Vivona N., Gulino A., D’Alessandro N., Notarbartolo M. (2015). Epigenetic changes and nuclear factor-κB activation, but not microRNA-224, downregulate Raf-1 kinase inhibitor protein in triple-negative breast cancer SUM 159 cells. Oncol. Lett..

[138] Boukerche H., Aissaoui H., Prévost C., Hirbec H., Das S.K., Su Z.Z., Sarkar D., Fisher P.B. (2010). Src kinase activation is mandatory for MDA-9/syntenin-mediated activation of nuclear factor-kappaB. Oncogene.

[139] Das S.K., Bhutia S.K., Sokhi U.K., Azab B., Su Z.Z., Boukerche H., Anwar T., Moen E.L., Chatterjee D., Pellecchia M. (2012). Raf kinase inhibitor RKIP inhibits MDA-9/syntenin-mediated metastasis in melanoma. Cancer Res..

[140] Notarbartolo M., Labbozzetta M., Pojero F., D’Alessandro N., Poma P. (2018). Potential Therapeutic Applications of MDA-9/Syntenin-NF-κB-RKIP Loop in Human Liver Carcinoma. Curr. Mol. Med..

[141] Pires B.R.B., Silva R.C.M.C., Ferreira G.M., Abdelhay E. (2018). NF-kappaB: Two Sides of the Same Coin. Genes (Basel).

[142] Dalgleish A.G. (2015). Rationale for combining immunotherapy with chemotherapy. Immunotherapy.

[143] Emens L.A., Middleton G. (2015). The interplay of immunotherapy and chemotherapy: Harnessing potential synergies. Cancer Immunol. Res..

[144] Formenti S.C., Demaria S. (2013). Combining radiotherapy and cancer immunotherapy: A paradigm shift. J. Natl. Cancer Inst..

